# SAW Hydrogen Sensors with Pd/SnO_2_ Layers

**DOI:** 10.3390/ma15228012

**Published:** 2022-11-13

**Authors:** Izabela Constantinoiu, Dana Miu, Cristian Viespe

**Affiliations:** 1 Laser Department, National Institute for Laser, Plasma and Radiation Physics, Atomistilor 409, RO-077125 Magurele, Romania; 2 Faculty of Chemical Engineering and Biotechnologies, University Politehnica of Bucharest, RO-011061 Bucharest, Romania

**Keywords:** pulsed laser deposition (PLD), surface acoustic wave (SAW), Pd, SnO_2_, bilayer, hydrogen, thin film, gas sensor, porous morphology

## Abstract

Pd/SnO_2_ bilayers for surface acoustic wave (SAW) sensors were obtained using pulsed laser deposition (PLD). Bilayers were made at several deposition pressures in order to observe the influence of the morphology of the sensitive films on the response of the sensors. The morphological properties were analyzed by scanning electron microscopy (SEM). The SnO_2_ monolayers were initially deposited on quartz substrates at 100, 400 and 700 mTorr, to observe their morphology at these pressures. The Pd/SnO_2_ bilayer depositions were made at 100 and 700 mTorr. The sensors realized with these sensitive films were tested at different hydrogen concentrations, in the range of 0.2–2%, at room temperature. In order to establish selectivity, tests for hydrogen, nitrogen, oxygen and carbon dioxide were carried out with SnO_2_-700, Pd-100/SnO_2_-700 and Pd-700/SnO_2_-700 sensors. The sensor with the most porous sensitive film (both films deposited at 700 mTorr) had the best results: a sensitivity of 0.21 Hz/ppm and a limit of detection (LOD) of 142 ppm. The morphology of the SnO_2_ film is the one that has the major influence on the sensor results, to the detriment of the Pd morphology. The use of Pd as a catalyst for hydrogen improved the sensitivity of the film considerably and the selectivity of the sensors for hydrogen.

## 1. Introduction

Hydrogen is considered a viable alternative for replacing fossil fuels, which have harmful effects on the environment [1]. However, for hydrogen applications in the automotive field and beyond, it is necessary to take safety measures, since at concentrations over 4% it becomes flammable and explosive [1,2]. Hydrogen is an odorless and colorless gas, which makes it difficult to detect [2,3,4]. Also, hydrogen gas leaks easily, its inflammability being explained by its ultra-small molecule size and high chemical activity [3]. Consequently, the control of hydrogen concentration in the installations that use it, as well as in surrounding areas, is an important aspect for its integration as a fuel. There is a need for devices such as sensors, which can monitor the concentration of hydrogen where it is likely to accumulate, keeping its concentration as low as possible and thus avoiding dangerous incidents [5].

A wide range of sensors for hydrogen detection have been developed to date: resistive, conductometric, chemoresistive, metal-oxide semiconductor, surface acoustic wave (SAW) sensors, etc. [6,7,8,9,10]. Each of these types of sensor has advantages and disadvantages that influence important parameters such as sensitivity, limit of detection (LOD) and response time, as well as costs of production and consumption. 

Surface acoustic wave sensors stand out due to their good sensitivity, satisfactory stability, possibility of wireless operation, ease of manufacture and small size [3,11,12]. They were developed both for hydrogen and for other gases such as volatile organic compounds, ammonia and other toxic and explosive substances [11,13,14]. A SAW sensor consists in a piezoelectric substrate, two pairs of interdigital transducers, and a sensitive film placed between the interdigital transducers [5,15]. Their operating principle is based on converting an electrical input into mechanical waves, which propagate over the sensitive film surface, and are subsequently converted back into an electrical signal. In the presence of the analyte at the surface of the sensitive film, a shift in the frequency of the waves occurs, due mainly to mass accumulations or acoustoelectric interactions. Thus, the nature and the type of sensitive film are very important aspects in the design of a SAW sensor for a certain type of gas [5,15]. 

Semiconductor metal oxides are widely used in sensors, including hydrogen sensors. Among the advantages they present in this domain are high sensitivity due to the wide bandgap, the possibility of miniaturization, reliability and ease of processing into sensor devices [16]. ZnO, TiO_2_, SnO_2_, WO_3_, In_2_O_3_, are some of the semiconductor oxides with very good sensing properties [16,17,18]. 

SnO_2_ is well known for its sensing properties, making it one of the most used oxides in this field [19]. This results from the fact that it is an n-type wide band-gap semiconductor (Eg = 3.6 eV at 300 K), with excellent electrical properties and chemical stability [17,20]. Some of the disadvantages of SnO_2_ are the difficulty of room-temperature detection, and the lack of selectivity, since it has very good sensitivity to a wide range of materials [2,19]. One of the methods used to obtain results with a SnO_2_ sensor at the lowest possible concentrations is to heat the sensor and use the temperature as an energy source to increase the interaction between the film and the analyte [21]. However, this method requires a heat source. In addition, detecting hydrogen at high temperatures becomes dangerous, since it can trigger an explosion at high hydrogen concentrations. Considering previous experience in the development of sensitive materials for hydrogen detection and other results from the literature, we found that optimizing the morphology of sensitive films for sensors (in this case, SAW sensors), leads to obtaining sensors capable of detecting hydrogen at room temperature [20,22,23]. This method is based on the principle of increasing the interaction surface between the analyte and the sensitive layer [19,20]. The larger the interaction surface, the lower the LOD and the shorter the response time [2,24]. 

The very low selectivity of SnO_2_ can be compensated by combining it with a selective material [2]. In the case of hydrogen detection, it is known that metals such as Pd or Pt have catalytic properties for hydrogen [25,26,27,28,29]. Thus, in their presence, the hydrogen molecule is dissociated and its subsequent penetration into the volume of the film is favored [16,30]. 

One morphology suitable for obtaining good sensitivity is the porous one [2,31,32]. Pulsed laser ablation (PLD) is a deposition method allowing good control of the morphology of the deposited film [22,33]. Pulsed laser ablation permits the control of many deposition parameters: deposition pressure, laser wavelength and power, pulse repetition rate, substrate temperature, target-substrate distance, etc. [33,34]. Among the other advantages of this method are maintaining the target stoichiometry in the deposited film, the possibility of making deposits from a large category of materials and at low temperatures, ensuring purity and good film adhesion [35,36,37]. Taking these considerations into account, PLD becomes a suitable method for developing hydrogen SAW sensors with optimal porous morphology. 

In previous work, we developed SAW sensors for hydrogen using PLD with different types of sensitive materials: ZnO, TiO_2_, WO_3_, Pd [18,22,23]. All these sensors were tested with good results at room temperature. It could be observed that in order to obtain sensibility for hydrogen, Pd/oxide (ZnO, TiO_2_, WO_3_) bilayer sensors have much better sensitivity and detection limits than sensors with monolayer oxide film. This improvement comes from the use of Pd as a catalyst layer for hydrogen molecules. We also observed that the porous morphology of the sensitive film improves the response of the sensors, offering better sensitivity and a shorter response time, based on the previously discussed principle [18,22,23]. Based on the above-mentioned results, and on the very good sensor properties of SnO_2_ presented in the literature, we proposed to develop SAW sensors for hydrogen with a bilayer structure of Pd and SnO_2_. 

The present paper presents research on the behavior of SnO_2_ and Pd in different PLD deposition conditions, and the influence of these conditions on SAW sensor results in hydrogen detection. The contribution of Pd to the improvement of the sensibility and selectivity of the SAW sensors at different gas concentrations was also investigated.

## 2. Materials and Methods

### 2.1. Film Deposition and Characterization

The sensitive films were deposited using the PLD method. A Nd-YVO4 laser was used, at stable parameters of 10 ps pulse duration, 0.2 W laser power and a repetition rate of 10 kHz. 

In order to study the effect of various parameters on the morphology of the sensitive film, the depositions were initially made onto quartz substrates (without interdigital transducers). Commercial SnO_2_ and Pd targets were used (Goodfellow, Huntingdon, UK), which were placed on computer-controlled x-y tables whose movement avoided target erosion. The substrates were placed parallel to the target, at a distance of 4 cm; the depositions were made at room temperature. The sensor substrate was positioned in a special mask, in order to limit the film deposition to the area between the interdigital transducers, and protect them from the deposition process. The depositions were carried out in two types of gas: oxygen for SnO_2_, and argon for Pd. Their pressure and flow were controlled by means of a system attached to the PLD deposition chamber. A throttle valve (MKS 253B) controlled by a pressure controller (MKS 600) mounted on a preliminary vacuum pump (Agilent Varian—DS602, Leini, Italy) adjusts the deposition pressure, and a mass-flow control system (MKS multigas 647, Munchen, Germany) controls the flow from the gas cylinders. The sensors and the deposition conditions are given in Table 1. The morphology of the films was analyzed by scanning electron microscopy (SEM) using a FEI QUANTA microscope (Hillsboro, OR, USA).

### 2.2. Sensor Structure and Testing

**Figure 1:**
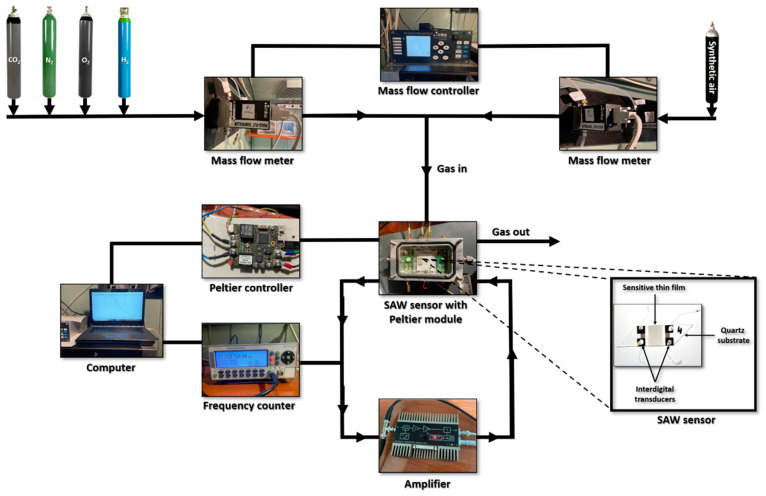
Experimental setup for SAW sensor measurements.

A ST-X quartz substrate was used for the SAW sensor, with a parallelogram geometry, in order to reduce unwanted reflections. There are several types of piezoelectric materials used for SAW sensors, but one advantage of the quartz substrate is its relatively low temperature coefficient in comparison to other piezoelectric materials [15]. The dimensions of the piezoelectric quartz are: 38 mm long, 10 mm wide and 0.5 mm thick. The SAW sensor is a delay-line type with the gold interdigital transducers in a double-comb configuration fabricated by photolithography. The oscillation frequency of the SAW sensors is about 69 MHz. The entire test system is presented in Figure 1. The oscillating system consists in a DHPVA amplifier (MessTechnik GmbH, Berlin, Germany) connected to a CNT-91 Pendulum frequency counter (Spectracom Corp, Rochester, NY, USA). The SAW sensors were tested at gas concentrations between 0.2% and 2%. For testing, we used a system consisting of gas cylinders, one with 100% artificial air, and the others with carbon dioxide (CO_2_), nitrogen (N_2_), oxygen (O_2_) and 2% H_2_ in synthetic air. The gas concentration in the test chamber was controlled with two mass flow meters connected to a mass flow controller. Throughout the experiments, the gas flow from the cylinders was maintained at a value of 0.5 L/min. The tests were initially carried out at room temperature, but tests were also carried out at different temperatures of the sensors. The temperature was varied in the range of 20–60 °C. The test system consisted in a Peltier element (70 W), bidirectional thermoelectric controller (TEC-1089-SV), temperature sensor for support (Pt100), computer, software interface, voltage source. This assembly allows the sensor temperature to be controlled with a precision greater than 0.01 °C.

## 3. Results and Discussion

### 3.1. Film Morphology

**Figure 2:**
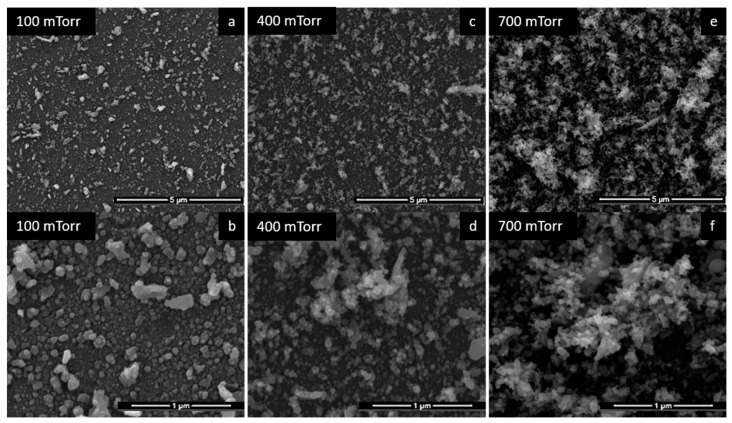
SEM images of SnO_2_ films deposited at different gas pressures.

Initially, the SnO_2_ and Pd monolayer films were analyzed through SEM images. The variation of the gas pressure (100, 400 and 700 mTorr) during the PLD deposition of the SnO_2_ thin films for 2 h led to different film morphologies. The SEM images in Figure 2 show the results of the variation of deposition gas pressure on the SnO_2_ layer. The increase in the degree of porosity with the increase in the deposition pressure can be observed. At the pressure of 100 mTorr (Figure 2a,b), the film is denser and presents granules of different shapes and sizes on the surface; however, these can induce a high level of noise in the sensors. Increasing the pressure to 400 mTorr (Figure 2c,d), the granules become smaller and are more numerous. When the gas pressure is higher, the interactions between the species in the plasma and the gas molecules are more pronounced. In these conditions, increased gas-phase nucleation occurs in the region between the target and the substrate. Increasing the pressure up to 700 mTorr, the morphology becomes much more porous, which is visible even at low magnifications of the SEM images (Figure 2e). It consists of spherical granules with dimensions of tens to hundreds of nm. A sensor with such morphology is expected to have the best results. The explanation is related to the fact that the more porous morphology implies a larger specific surface, therefore a larger surface of interaction between the analyte molecules and the sensitive film [2,3,4,5,6,7,8,9,10,11,12,13,14,15,16,17,18,19,20,21,22,23,24]. Thus, detection can be performed at lower concentrations, with a shorter response time and at room temperature.

**Figure 3:**
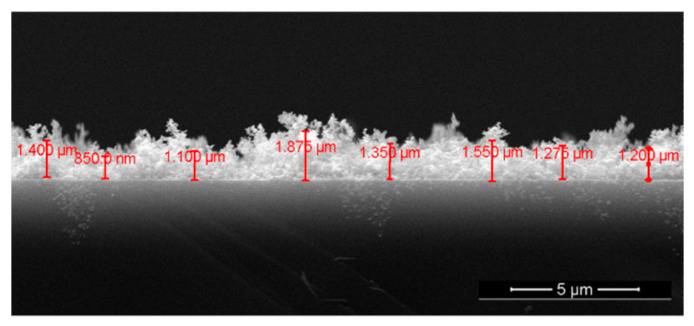
Cross-section SEM image of the SnO_2_ film deposited at 700 mTorr.

The high porosity of the film deposited at 700 mTorr is confirmed by the cross-section SEM image in Figure 3. From the measurements made on several SEM cross-section images, the average thickness of the SnO_2_ film deposited at 700 mTorr oxygen was approximately 1400 nm.

**Figure 4:**
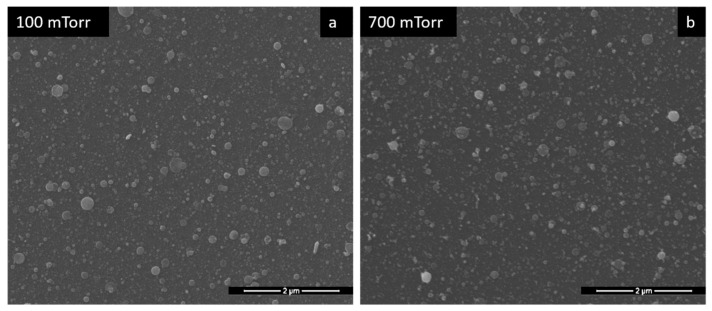
SEM images of Pd films at different gas pressures.

The role of Pd in the bilayer structure of the sensitive film is as a catalyst for hydrogen molecules, thus improving both the sensitivity and the selectivity of the sensor. It was found that with the increase in the thickness of the Pd film, its catalytic activity on hydrogen molecules is decreased [3]. Therefore, the Pd layer must have a very small thickness, compared to the sensitive base layer. Thus, in the present case, PLD depositions of 2′23″ were carried out, at 100 and 700 mTorr, to observe the influence of pressure on Pd morphology. Both SEM images in Figure 4 of Pd layers, described above, indicate the formation of continuous Pd layers with spherical formations of different sizes on the surface. No major difference can be identified between 100 and 700 mTorr deposition pressure, considering the very small amount of deposited material. Therefore, regarding the performance of the sensors depending on the Pd deposition pressure, we did not expect that the Pd deposition pressure would have a significant influence. 

**Figure 5:**
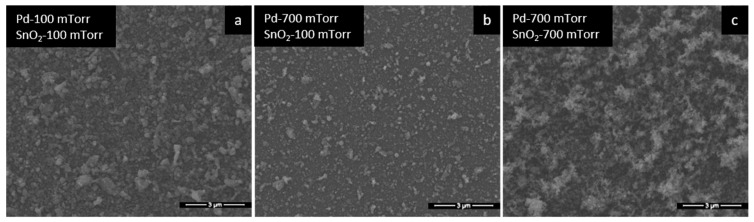
SEM images of Pd/SnO_2_ films at different gas pressures.

The bilayer films were deposited at 100 and 700 mTorr, both for SnO_2_ and Pd (Table 1). These pressures were chosen because they form considerably different morphologies, so that the influence of the morphology on the sensor results can be highlighted. Comparing the SEM images from Figure 2 (images with only SnO_2_ films), with the corresponding ones from Figure 5 (only with bilayer films), we notice that the morphology is similar. Thus, the presence of Pd is not decisive for a change in the morphology of the bilayer films, neither at 100 mTorr nor at 700 mTorr. This is due to the small amount of Pd deposited on the surface of the SnO_2_ films, as explained previously. In conclusion, we observe that the morphology of Pd/SnO_2_ bilayer thin films is dictated by the morphology of SnO_2_ films.

### 3.2. Sensor Properties

Following the tests carried out at different hydrogen concentrations, all the sensors responded at room temperature. The tests were carried out at hydrogen concentrations between 0.2 and 2%. Taking into account that over a concentration of 4% hydrogen becomes flammable and explosive, the sensors must be able to detect lower concentrations. 

**Figure 6:**
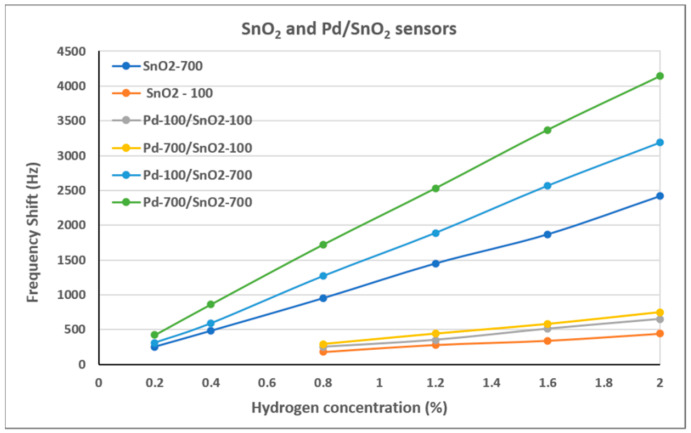
Frequency shift of the SnO_2_ and Pd/SnO_2_ sensors at different hydrogen concentrations.

Figure 6 shows the frequency shifts of the SAW sensors tested at different hydrogen concentrations. It can be observed that there is a clear delimitation between the sensor results for SnO_2_ films deposited at different pressures. The sensors with the SnO_2_ films deposited at 700 mTorr (more porous) recorded frequency shifts approximately six times higher than those with SnO_2_ deposited at 100 mTorr for the same hydrogen concentration. This confirms that the morphology of SnO_2_ is the major influence on the sensor results. However, the presence of Pd also makes a difference in the sensor results. It can be observed that both bilayer films with SnO_2_ films deposited at 700 mTorr (Pd-100/SnO_2_-700 and Pd-700/SnO_2_-700) led to better results than the single layer SnO_2_-700 sensor. The same observation is true for sensors with SnO_2_ deposited at 100 mTorr. However, there is a relatively small difference between the sensors with different deposition pressures of the Pd films, those with Pd deposited at 700 mTorr having a slightly higher frequency shift than those deposited at 100 mTorr.

An important aspect in the characterization of sensors is selectivity. When the sensitive material of the sensor has sensitive properties for several types of gases, it is necessary to use a selective element for the gas of interest. Semiconductor metal oxides, especially SnO_2_, are good sensing materials for a wide range of gases. 

**Figure 7:**
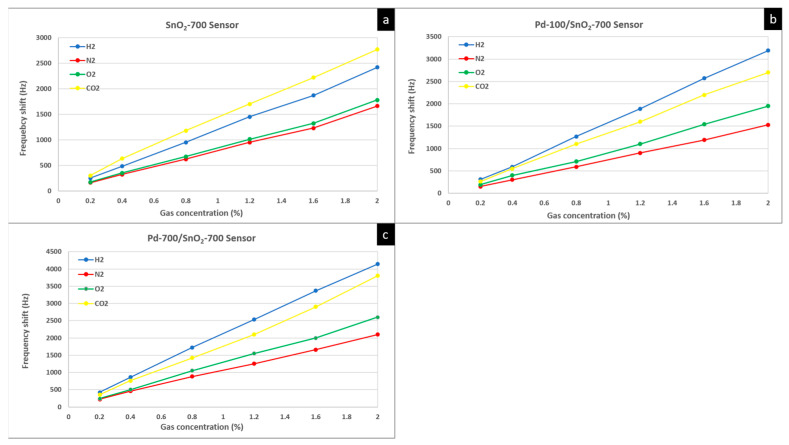
Frequency shifts of: (**a**) SnO_2_ sensor, (**b**) Pd-100/SnO_2_-700 sensor and (**c**) Pd-700/SnO_2_-700 sensor, at different concentrations of H_2_, N_2_, O_2_ and CO_2_.

In order to determine the degree of selectivity, the three sensors that obtained the best hydrogen sensitivity (SnO_2_-700 sensor, Pd-100/SnO_2_-700 sensor and SnO_2_-700/Pd-700 sensor) were chosen for tests on several gases: hydrogen (H_2_), nitrogen (N_2_), oxygen (O_2_) and carbon dioxide (CO_2_). The frequency shifts recorded by these sensors are presented in the graphs in Figure 7. All sensors had results in the gas concentration range of 0.2–2%. 

The tests showed that the best result of the SnO_2_-700 sensor was for CO_2_ gas. The Pd-100-SnO_2_-700 sensor and Pd-700/SnO_2_-700 sensor, on the other hand, had the best results for hydrogen. The major difference between the mentioned sensors is given by the presence of Pd. Thus, we can state that the use of Pd represents an important point for obtaining sensors with an important degree of hydrogen selectivity. The mechanism of action of Pd was explained previously, and through these tests it was confirmed. 

**Figure 8:**
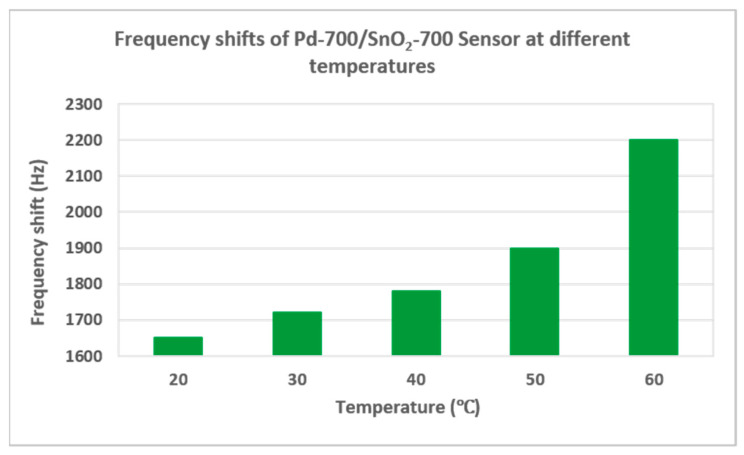
Frequency shifts of Pd-700/SnO_2_-700 sensor, at different temperatures, for a concentration of 0.8% hydrogen.

For a wide category of sensors, the use of thermal energy during detection improves sensor properties. The tests were carried out after the sensors reached the set temperature, which was kept constant with a Peltier controller. According to the graph in Figure 8, as the sensor temperature increases, the frequency shift is greater. In addition, considering the pronounced danger of explosion that hydrogen presents at an accumulation of only 4% in the environment, the temperature factor coming from the sensor can be the catalyst of the explosion reaction in an emergency situation, because the reactivity increases with temperature. 

**Figure 9:**
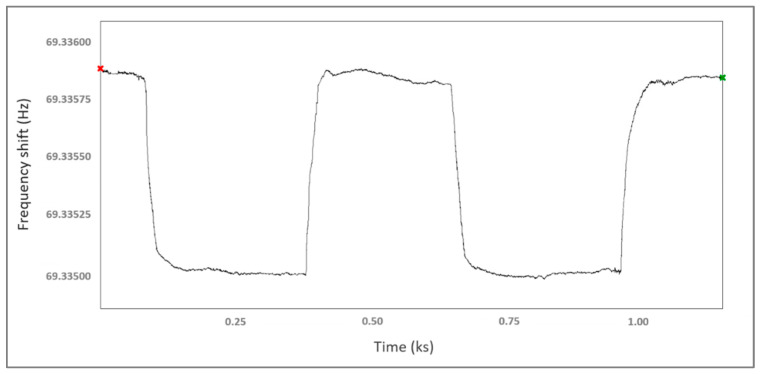
Dynamic response of sensor Pd-700/SnO_2_-700 at 0.4% hydrogen concentration.

One of the important advantages of SAW sensors is repeatability. This is ensured by using a sensitive film that does not irreversibly interact with the gas molecules, allowing their desorption. The sensitive films obtained in this work present this property, the reversibility being proven by the dynamic response of the Pd-700/SnO_2_-700 sensor in Figure 9, at 0.4% hydrogen concentration. Ten consecutive measurements were made for Pd-700/SnO_2_-700 and Pd-100/SnO_2_-700 sensors. After the tests, a relative error of ±4% was determined. From the same graph, the response and recovery times were calculated, which were approximately 24 s and 30 s, respectively. The intervals of these times for all sensors were 20–30 s for the response time and 25–40 s for the recovery time. 

The sensitivity is defined as the frequency shift in Hz per unit analyte concentration in ppm; it was determined from an average sensitivity value of the determinations at each gas concentration [15]. The LOD is defined as three times the noise level divided by the sensitivity [15]. The noise level was estimated at 10 Hz. Table 2 shows the results of the calculations for sensitivity and LOD for all sensors. It can be observed that the best results were obtained by the sensors with sensitive films deposited at 700 mTorr (more porous), both for SnO_2_ and Pd (Pd-700/SnO_2_-700). These results are comparable or better than other results from the literature and from our previous experiments [18,22,23,38,39]. In our previous work [22], we developed Pd/TiO_2_ sensors for hydrogen detection. The best sensitivity obtained in that case was of 0.10 Hz/ppm, with the sensor with the highest porosity in those experiments, which was two times less than the best sensitivity obtained in this work (0.21 Hz/ppm). The LOD for this best Pd/TiO_2_ sensor was 1210 ppm, eight times higher than the one obtained in the present work for Pd/SnO_2_ (142 ppm). An important factor for this result is the much lower level of noise recorded in the present work (10 Hz, compared to 40 Hz in [22]).

These results are a consequence of the mechanism of interactions between the sensitive film and the hydrogen molecules. The mechanism consists in several stages, as shown in the equations below (R1–R8) [3]. First of all, when the sensor is exposed to air, oxygen molecules accumulate at the level of the sensitive film. Upon their interaction with Pd, they are dissociated into oxygen atoms, which will capture electrons from the SnO_2_ layer and become species such as $O^{-},{\;O}^{2-},{\;O}_{2}^{-}$. They spill over to the SnO_2_ film and accumulate at the intergranular boundaries.

(1) $$O_{2\left(g\right)}+\mathrm{Pd}\to 2O\left(\mathrm{Pd}\right)\;\left(\mathrm{dissociation}\right)$$

(2) $$O_{2\left(g\right)}+O\left(\mathrm{Pd}\right)\to O_{\left(\mathrm{surf}\right)}\left({\mathrm{SnO}}_{2}\right)+2O\left(\mathrm{Pd}\right)\;\left(\mathrm{spillover}\right)$$

When hydrogen molecules appear at the level of the sensitive film, some of them react with the oxygen species accumulated at the level of the sensitive film, forming water molecules in vapor state. The rest of the hydrogen molecules are dissociated upon interaction with Pd into atomic hydrogen and spill over to the SnO_2_. At this point, hydrogen atoms and oxygen species adsorbed at the SnO_2_ layer interact, forming water vapor.

(3) $$H_{2\left(g\right)}+O\left(\mathrm{Pd}\right)\to H_{2}O_{g}↑+\left(\mathrm{Pd}\right)$$

(4) $$H_{2\left(g\right)}\to H+H\;\left(\mathrm{dissociation}\right)$$

(5) $$H+{\mathrm{SnO}}_{2}\to H_{\mathrm{ad}}\left({\mathrm{SnO}}_{2}\right)\;\left(\mathrm{spillover}\right)$$

(6) $$H_{\mathrm{ad}}\left({\mathrm{SnO}}_{2}\right)+O_{\mathrm{ad}}^{-}\left({\mathrm{SnO}}_{2}\right)\to {\mathrm{OH}}_{\mathrm{ad}}^{-}\left({\mathrm{SnO}}_{2}\right)$$

(7) $${\mathrm{OH}}_{\mathrm{ad}}^{-}\left({\mathrm{SnO}}_{2}\right)+H_{\mathrm{ad}}\left({\mathrm{SnO}}_{2}\right)\to H_{2}O\left(g\right)↑+\left({\mathrm{SnO}}_{2}\right)+e^{-}$$

(8) $$2H_{\mathrm{ad}}\left({\mathrm{SnO}}_{2}\right)+O_{\mathrm{ad}}^{2-}\left({\mathrm{SnO}}_{2}\right)\to H_{2}O\left(g\right)↑+\left({\mathrm{SnO}}_{2}\right)+2e^{-}\;$$

According to the reactions above, this whole process leads to the release of electrons in the conduction band of SnO_2_, thus increasing its conductivity. This increase in conductivity slows the speed of the SAW waves and reduces the central frequency of the gas sensor. Although the interaction that causes the change of SnO_2_ conductivity takes place at the SnO_2_ film, the presence of the Pd layer plays a role as well. The SnO_2_-modified layer with Pd leads to an improvement of its sensitivity, by facilitating the interaction of hydrogen with oxygen species, resulting in changes in the conductivity of the film. It is very important to monitor the uniformity of the Pd film and, of course, its thickness, to allow the complete dissociation reaction.

## 4. Summary and Conclusions

Thin films of SnO_2_ and Pd/SnO_2_ were deposited by PLD onto quartz substrates. Different pressures of oxygen and argon were used for depositing SnO_2_ and Pd, respectively, and their influence on the morphology of the film was discussed based on SEM images. It was observed that as pressure increases, the degree of porosity of the films increases. Surface acoustic wave sensors were realized with single layer SnO_2_ and bilayer Pd/SnO_2_ sensitive films with different morphologies, deposited at 100 and 700 mTorr. The sensors were tested at different hydrogen concentrations (0.2–2%) at room temperature. The best results were obtained for the sensor with a bilayer sensitive film, when the depositions were made at 700 mTorr, both in the case of SnO_2_ and in the case of Pd. The sensitivity of this sensor (Pd-700/SnO_2_-700) was 0.21 Hz/ppm, with a LOD of 142 ppm. The weakest result was of the sensor with only SnO_2_ film, deposited at 100 mTorr. Thus, we see the importance of both the morphology and the presence of Pd in order to obtain sensors with the best possible results. The importance of Pd in the detection of hydrogen was also highlighted after the tests on several gases, when the best result of the sensor without Pd was for CO_2_ and the best results for the sensors with Pd were for hydrogen. Tests at temperatures in the 20–60 ℃ range showed an increase in the frequency shift with the increase in temperature, but not with a considerable value. 

These results were better than previous results obtained for sensors with Pd/TiO_2_ layers (LOD for this best Pd/TiO_2_ sensor was 1210 ppm, eight times higher than the one obtained in the present work for Pd/SnO_2_ (142 ppm)) [22] and represent a good and important direction of study for the development of SAW sensors with better performances. Also, the importance of Pd and its influence depending on the thickness for such structures represents a subject of interest for study. 

## Figures and Tables

**Table 1:** The sensors and the thin film deposition characteristics.

Sensor	Composition of the Sensitive Layer	Deposition Pressure (mTorr)	
		O_2_	Ar
SnO_2_-100	SnO_2_	100	-
SnO_2_-700		700	-
Pd-100/SnO_2_-100	Pd/SnO_2_	100	100
Pd-100/SnO_2_-700		700	100
Pd-700/SnO_2_-100		100	700
Pd-700/SnO_2_-700		700	700

**Table 2:** Sensitivity and limit of detection (LOD) of the sensors (Δf-frequency change; c-hydrogen concentration, n-noise level).

Sensor	Sensitivity (Δf/c) (Hz/ppm)	LOD (3 × *n*)/(Δf/c) (ppm)
SnO_2_-100	0.02	1349
SnO_2_-700	0.12	251
Pd-100/SnO_2_-100	0.03	963
Pd-100/SnO_2_-700	0.16	189
Pd-700/SnO_2_-100	0.04	818
Pd-700/SnO_2_-700	0.21	142
